# Wearables for the Next Pandemic

**DOI:** 10.1109/ACCESS.2020.3029130

**Published:** 2020-10-06

**Authors:** Ava Hedayatipour, Nicole Mcfarlane

**Affiliations:** 1 Department of Electrical EngineeringCalifornia State University Long Beach CA 90840 USA; 2 Department of Electrical Engineering and Computer ScienceThe University of Tennessee 4285 Knoxville TN 37996 USA

**Keywords:** Bioimpedance, circuits and systems, COVID-19, influenza, pandemic, pulse oximetry, sensors, temperature sensor, wearable devices

## Abstract

This paper reviews the current state of the art in wearable sensors, including current challenges, that can alleviate the loads on hospitals and medical centers. During the COVID-19 Pandemic in 2020, healthcare systems were overwhelmed by people with mild to severe symptoms needing care. A careful study of pandemics and their symptoms in the past 100 years reveals common traits that should be monitored for managing the health and economic costs. Cheap, low power, and portable multi-modal-sensors that detect the common symptoms can be stockpiled and ready for the next pandemic. These sensors include temperature sensors for fever monitoring, pulse oximetry sensors for blood oxygen levels, impedance sensors for thoracic impedance, and other state sensors that can be integrated into a single system and connected to a smartphone or data center. Both research and commercial medically approved devices are reviewed with an emphasis on the electronics required to realize the sensing. The performance characteristics, such as accuracy, power, resolution, and size of each sensor modality are critically examined. A discussion of the characteristics, research challenges, and features of an ideal integrated wearable system is also presented.

## I. Introduction

This year marked the start of a global pandemic that has caused 22 million unemployment in the United States alone and can cost the global economy up to 4.7 trillion dollars [1]. With a history as old as humanity, humankind has been dealing with contagious diseases. The Plague of Justinian (541-542 with 30–50 million fatalities), Bubonic plague (1347-1351 with 200 million fatalities), Small Pox (1520 with 5 million fatalities), Great Plague ($17^{th}$-$18^{th}$ century with 16 million fatalities), Spanish flu (1918-1919 with 40–50 million fatalities) and HIV (1981-Present with 25–35 million fatalities) are among pandemics on record that have changed the course of history [2]. Humankind has held back these pandemics, and possibly our extinction, with the hard work and collaboration of many great healthcare workers, researchers, scientists, pathologists, and epidemiologists.

We faced a new challenge in 2020, a pandemic in the age of technology. In the $21^{st}$ century, the overall quality of life and life expectancy has increased considerably compared to the early $20^{th}$ century. One hundred years ago, the typical newborn fatality rate in the first year of life was 26.9% and 46.2% children born died before they reached adulthood [3]. In 1900 the typical life expectancy was 41 years, making the effect of pandemic mortality rate less on people’s lives. With the advancement of medicine, the modern scientific understanding of diseases, improved understanding of hygiene, timely treatment, and diagnosis of disease, the average life expectancy in the world has almost doubled [4], making the 2020 pandemic a world scale crisis.

We are still unsure of the human and economic recovery process. The first step of recovery is knowing when the pandemic is going to be over. Similar to many past pandemics, people are waiting for a vaccine to overturn the situation. In the middle of the $20^{th}$ century, vaccines went through a revolution that regenerated previous vaccines with specific antigens, making them more efficacious, safer, and less expensive. Growing viruses in laboratories led to the evolution of the now common childhood vaccination schedules for diseases such as poliomyelitis, measles, and whooping cough. In recent years of vaccine research, scientists have pursued new directions through innovative techniques using recombinant DNA and novel loading techniques [5], [6]. Vaccine research has expanded its target to various diseases such as hepatitis B, influenza, pneumococcus (which causes pneumonia), meningococcus (which causes meningitis), and septicemia. To go even further, researchers are targeting non-infectious conditions such as addictions and allergies.

Currently, most vaccines are derived from our knowledge of the immune response and host-pathogen interactions along with mass production of antigens. However, the speed of generating the vaccine has still remained lengthy, and it is estimated as of spring 2020 that a COVID-19 vaccine can take between 12–18 months to reach mass production [7]. Though making the vaccine is much faster and more reliable than the $20^{th}$ century, raised standards on the number of trials and human tests needed before a vaccine is mass-produced keeps the timing slower or the same as vaccine production for previous pandemics. Here are the facts to take away, similar to previous pandemics, the vaccine will be able to help after the disease has reached its peak.

Unlike vaccines, communication technology has changed drastically since the pandemics in the early $20^{th}$ century. In 1920 the first radio channel was broadcast and in 1925, the first television signals were broadcast [8]. Thus, people have been more informed during a pandemic. Reading the history of pandemics, one can notice that before such communication, each community needed to identify a pandemic on their own, leading to delay in diagnosis in many areas, which caused more severe outcomes in terms of fatality and infection rate. Moreover, the right approaches to control the contagion and possible vaccine, cures, and information regarding the disease also took a lot of time to reach each community. Individuals were bearing the load of inventing the wheel over and over again, often halted even more with the misinformation that a letter or a short telegram would bear. An example is the 1918 Spanish flu when these messages were carried by postal workers and taught to the public by postal workers, boy scouts, and teachers. One goal of this campaign was to prevent people from using shared cups at water fountains or to remove paper towels from restrooms. Similar to the Covid-19 lockdowns in many countries, these volunteers were also used to provide food to highly impacted communities [9].

Despite a few similarities, in our time, the abundance of communication technologies has allowed us to stay in contact with friends, family, and social media news hubs along with older means of information (radio, television, newspaper). We are bombarded with news every day, it is reported by WHO that a surge of unconfirmed news can lead to losing the public’s trust with severe effects on health, the economy, and political terms [10]. However, the abundance of technological devices available and the prevalence of wearable devices are what separate the current era from the past. It is up to us to put these technologies to good and efficient use.

This paper outlines the roles of portable wearable devices in controlling pandemics and aims to discuss the advantages and limitations of incorporating $21^{st}$-century technologies (wearable medical systems, mobile devices) in patient monitoring. After the introduction, a brief history of pandemics along with their symptoms is presented in Section II. Section III goes through the different technologies available today for pandemics and what has been done to monitor previous pandemics. Different sensors that can help detect respiratory pandemics symptoms currently being researched or available commercially are reviewed in Section IV. In section V the feasibility and challenges of having portable sensors for pandemics is discussed, and finally the conclusion is given in section VI.

## II. Understanding Pandemics and Their Symptoms

**FIGURE 1.**
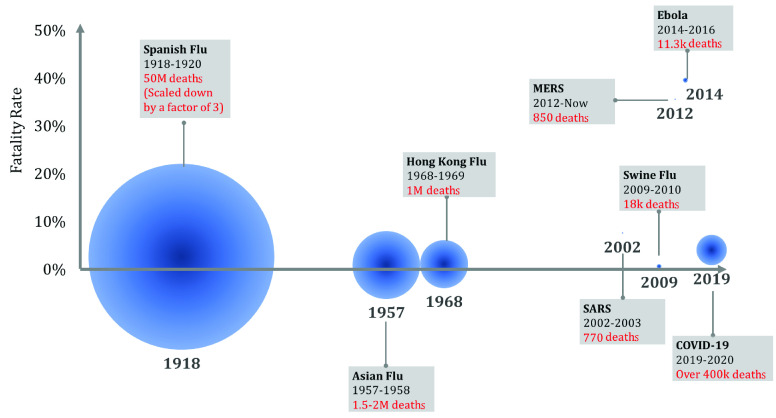
A timeline of the pandemics in the last 100 years. The death toll of each disease is represented by the area of the circle.

To have a better understanding, we go through the pandemics of the last 100 years. An illustration of the pandemics, a timeline of their year, death toll, and mortality rate is shown in Fig. 1. The 1918 influenza pandemic, **Spanish flu**, is still one of the most severe in recent times. This virus had an avian origin and was caused by an H1N1 virus. Though called the Spanish flu, researchers are still unsure of the birthplace of the virus. France, China, Britain, and the United States have all been suggested as a potential origin of the virus. It is estimated that at the time, 500 million people (roughly 1/3 of the global population) were infected by this virus [11]. With around a 10% mortality rate, especially in young adults (20-40 years) this virus is still one of the deadliest and most devastating in our recent history. Having no vaccine and no antibiotics to protect against secondary microbial infections caused by the influenza were among the contributing parameters to fatality of this pandemic. Quarantine, limitation of public gathering, and isolation of sick people were applied by governments (however unevenly). However, lack of appropriate personal hygiene and effective disinfectants can be associated with Spanish flu high mortality. After following its natural curve, the virus disappeared with a rapid decline in cases in late 1919 [11].

The next influenza pandemic initiated in China in February 1957, known as the **Asian flu**, killed at least 1 million people worldwide. This influenza was caused by a strain of an H2N2 virus, which was a mixture of avian and human flu with minimum immunity in the population. With the advancement of medicine, a vaccine was available from October 1957 that helped to control the pandemic. This virus circulated until 1958 and had an antigenic shift into a influenza A virus subtype H3N2 that caused the next big epidemic in 1968 [12].

The **Hong Kong flu**, initiated in 1968, caused 1 million fatalities globally with approximately 100,000 fatalities in the United States alone. The disease was particularly deadly to people 65 years old and older or people with pre-existing conditions. The vaccine was made widely available after the pandemic had already peaked for most populations. Medical care was more advanced compared to the early $20^{th}$ century and an abundance of antibiotics could prevent fatality due to secondary infections caused by the virus resulting in a fatality ratio under 0.5% [13].

Though advancements in medicine, pathology, and virology, we are immune to many diseases that cast challenges in history. Contagious respiratory illnesses still remains a threat to our well being in the modern world. In March 2003, a new respiratory pneumonia was diagnosed with the first infected humans located in China, and a global alert on **severe acute respiratory syndrome (SARS)** was issued by the WHO. SARS affected people in 30 countries, infecting more than 8000 people. With a considerable mortality rate of 4–10%, 774 died. With the virus effects developing deep in the lungs, this made the mortality rate high. The same fact, however, made the disease less contagious and easier to contain. The economic impact worldwide was over US ${\$}30$ billion [14].

In April 2009, a **H1N1** virus emerged, with the first human being diagnosed in Mexico. The origin of this disease remains unclear since the mixture of influenza genes is not identified by whole in avian, humans, or barn animals. This virus swept the United States and the world with an estimated number of infected people between 700 million to 1.4 billion people from April 2009 to August 2010 [15]. This corresponds to 11% to 21% of the world’s population at the time, more than the number of cases estimated for Spanish flu. However, this disease had a minor mortality rate of 0.01–0.03% of those infected, resulting in about 150,000 to 575,000 fatalities. Immunity of approximately 1/3 of adults older than 60 years was an unusual feature of this pandemic [16].

The next pandemic was first identified in Saudi Arabia in mid-2012. This disease called **Middle East Respiratory Syndrome Coronavirus (MERS-CoV)** infected 26 countries in Asia, Europe, and Africa with more than 80% of its cases in Saudi Arabia. The still ongoing pandemic does not pass easily from person to person, most infections are reported in people providing unprotected care to an infected patient. With the case-fatality rate as high as 35%, a significant number of laboratory-confirmed cases and deaths have been reported. The median age of affected individuals is 56 years, ranging from 2–94 years, with symptoms appearing about 5 or 6 days after a person is exposed, and ranging from 2 to 14 days [17].

The last pandemic before COVID-19 was **Ebola**. The first case was identified in a small village in Guinea, with an 18-month-old boy reported being infected by bats. The disease is believed to have happened before in 1976. The largest outbreak, however, occurred December 2013 to January 2016, with 28,646 cases and 11,323 deaths [18]. Over the duration of the epidemic Ebola, with a high fatality rate of 39.5%, spread to seven more countries. The vaccine was not developed until December 2016. Since its approval, the vaccine has been stockpiled and is believed to have stopped another outbreak in 2018 [19]. The summary of key findings regarding each pandemic is shown in Table 1.

**TABLE 1:** Pandemics of the Last 100 Years

Disease	Year	Death Toll	Fatality Rate	Age Range	Symptom Duration	Symptom Onset	Virus
Spanish Flu	1918–1920	50 million	2%	20–40	N.A.	N.A.	H1N1
Asian Flu	1957–1958	1.5–2 million	0.30%	Immunity rare < 65 years	N.A.	N.A.	H2N2
Hong Kong Flu	1968–1969	1 million	0.50%	Over 65	4–5 days	1–2 days	H3N2
SARS	2002–2003	770	4–10%	Immunity rare < 65 years	14 days	10 days	SARS-CoV
Swine Flu (H1N1)	2009–2010	Over 18k	0.01–0.03%	25 years and older	8 days	3–5 days	Novel H1N1
MERS	2012-Now	850	35%	2–94 years	7–16 days	5–6 days	MERS-CoV
Ebola	2014–2016	11.3k	39%	25–45 years	6–16 days	2–21 days	Ebola Virus

Typical pandemics symptoms resemble with flu symptoms. In the first wave of the **Spanish flu**, sick individuals experienced fatigue, chills, and fever. This was usually overcome within a few days, and the death rate was relatively low. In a second, and highly contagious wave, infected persons developed symptoms such as turning blue and fluid filled lungs, and died within days or hours. Initial flu-like symptoms of the illness were followed by appetite loss, stomach issues, and a dry hacking cough. The leading cause of death was pneumonia or other respiratory complications [20]. The **Asian flu** symptoms presented similarly to other strains of influenza and complications included heart failure, pneumonia, and seizures potentially leading to death.

The **Hong Kong Flu** caused upper respiratory symptoms along with muscle pain, weakness, chills, and fever with pneumonia is the leading cause of death among high-risk patients. **SARS** usually initiates with flu-like signs. After about a week, dry cough and shortness of breath appear, and ten days after a person is exposed to the virus, breathing issues arise. The infection then affects the lungs and airways (respiratory system), leading to an increasing lack of oxygen in the blood, which can be fatal in the most severe cases.

H1N1 Influenza starts with typical influenza symptoms with complications of worsening any chronic conditions, such as heart disease, asthma, and pneumonia. Severe pneumonia effects, particularly in the elderly, ranges from confusion to seizures and respiratory failure. Most people confirmed to have MERS-CoV infection also showed respiratory impairment with fever, cough, and shortness of breath followed by severe complications such as pneumonia and kidney failure. Ebola symptoms typically start with a sudden flu-like disease with symptoms of fever, tiredness and general weakness, reduced appetite, muscle and joint pain, headaches, and sore throat. Infected patients then present with nausea, vomiting, diarrhea, and abdominal pain. In addition, shortness of breath, chest pain swelling, headaches, and confusion may also be present.

As for **COVID-19**, the symptoms match what is on record for every other respiratory pandemic. There is an incubation period of up to 14 days before developing symptoms. The most common symptoms are persistent dry cough, fever, and tiredness. Acute respiratory failure and pneumonia are among the complication that can cause fatality among patients. Though, as of today, an increasing fatality rate of 3.4% has been reported by WHO, most people recover treatment free [21]. However shortness of breath and low blood oxygen, common among most COVID-19 patients, is an uncomfortable experience pushing patients to visit a hospital.

A summary of symptoms associated with pandemics is illustrated in Table 2. Fever is the most common symptom between all of the epidemics. Low blood oxygen occurs in all of the pandemics but Ebola. As a third parameter, all of the patients with complications had infection reaching their lungs. These infections cause inflammation in the respiratory tree. The outpouring of inflammatory fluids into the lungs causes pneumonia. As fluid collects in the lungs, the amount of oxygen carried to blood decreases. Insufficient oxygen in the blood can cause the kidneys, lungs, liver, and other organs to shut down and stop working [22]. A wearable sensing system that can detect these three main symptoms (fever, low blood oxygen, and fluid accumulation in lungs) can be used to identify the progress of the disease.

**TABLE 2:** Symptoms of Last 100 Years of Pandemics

Disease	Fever	$SpO_{2}$	Pain	Fatality Cause
Spanish Flu^*^	Yes	Low	Muscle	Pneumonia
Asian Flu^*^	Yes	Low	Muscle	Pneumonia
HongKong Flu^*^	Yes	Low	Muscle	Pneumonia
SARS	$> 38.0~^\circ \text{C}$	$ < 92\%$	Muscle	Pneumonia
H1N1	$> 37.7~^\circ \text{C}$	$ < 90\%$	Muscle	Pneumonia
MERS	$> 38.0~^\circ \text{C}$	$ < 90\%$	Chest	Pneumonia Kidney Failure
Ebola	$> 38.3~^\circ \text{C}$	No	Muscle	Bleeding
Abdominal	Fluid Loss
COVID-19	$> 38.3~^\circ \text{C}$	$ < 90\%$	Muscle	Pneumonia

^*^ No fever degree or blood oxygen ($SpO_{2}$) on record since the disease is for more than 50 years ago.

## III. Technology for Pandemics

Technology plays a rapidly growing role in daily routines. People are increasingly dependent on technology and less willing to be separated from it. Technology’s tendency to become faster and smaller has given rise to a new generation of wearable technology, from fitness trackers and the smart watch to smart glasses and smart contact lenses. The last two decades have seen an unprecedented increase in wearable technology usage, especially for healthcare applications [23]. The global wearable technology market was at US $750 million in 2012 and is expected to increase to US $51.6 billion by 2022 [24]. 60.5 million people used a wearable device in the US in 2019 and this number is expected to go up 9.2% by 2021 [24].

COVID-19 has increased the global demand for in-home monitoring and provider-patient engagement solutions. Current WHO guidelines emphasize COVID-19 patient monitoring based on temperature, breathing rate, and blood oxygen content. Many companies and startups in the sensor technology area are adapting existing technology to meet this increased need. These companies deliver secure cloud-based solutions to monitor COVID-19 symptoms. However, these solutions proposed by companies developing wearable gadgets, only follow one or a couple of related symptoms. As a note, all devices and solutions introduced in this article approved by the US Food and Drug Administration (FDA). The author extracted the FDA report for each device. If a report was not found, news of the device being FDA approved in a trustful technology news website was considered if the company had a running website and was responsive to quote requests. Though sensors claiming to be an FDA-approved product are being sold in well-known online shopping websites like Amazon, only sensors with official FDA reports are considered. Moreover, sensors included had to have some means of data transfer to the cloud, cell phone, or a medical data center. As the last condition, all included sensors can be bought from the relevant company website.

Masimo’s solution [25] is a secure cloud-based patient monitoring with wireless pulse oximetry for oxygen saturation and respiration rate. An online data server, Masimo SafetyNet Data Capture, monitors the results. The sensor is single use. Patient data is securely transmitted via Bluetooth to the Masimo SafetyNet mobile application. A predefined CareProgram, based on a patient care plan, provides notification and captures information from the wireless sensor. The data is then sent securely to the hospital for evaluation. The care program created by Masimo is in line with CDC and WHO guidance for tracking potential COVID-19 patients. Changes in official guidance or hospital protocol can be easily reflected in updates to the program.

Vital Patch [26] is another wearable fabrication company that has proposed a solution for COVID-19. Instead of admitting all patients to the hospital, at home continuous monitoring at the patient’s home environment, using a wearable patch that lasts up to 7 days, is instead used. Changes in typical parameters such as temperature, pulse, oxygen levels, and respiratory rate can initiate customizable alerts to bring attention to any worsening of symptoms. A total of 11 vital signs, including posture and fall detection are monitored. The high price of the system with the tablet and data center move this device further from commercial in-home use and in the realm of medical/hospital use.

Oxitone [27] points out the unique challenge the novel Coronavirus or COVID-19 infection imposes on us. According to their website, wearable real-time continuous digital monitoring of vital health signs is valuable in the fight against COVID-19. Symptoms in infected patients can range from none to mild to severe. However, one of the most dangerous symptoms is low oxygen levels in your blood without any other symptoms. These low levels of oxygen can cause serious damage to different organs like the heart, brain, or kidney. Using Oxitone, people with known or suspected exposure to COVID-19 can know their blood oxygen, temperature, and other vital parameters monitored from home. Oxitone can also monitor stress levels and sleep cycles during current circumstances when people are more prone to anxiety or depression.

Oxitone offers wearable technologies that fit COVID-19 home monitoring needs. Unlike many consumer-grade devices, these wearable solutions enable hospital-grade continuous measurements of the pulse oximetry data, heart rate, activity, stress level, sleep and stress pattern, skin temperature, and other parameters - all in one device. However, the system lacks respiratory rate detection. It was announced by the company that respiratory rate detection will be added to this sensor in the subsequent stages, along with fall detection. All the data is utilized to generate a unique personalized digital health signature. The system provides a physicians’ web portal with a secured cloud infrastructure for remote monitoring of captured data. Oxitone 1000M is FDA-cleared and the wrist is used instead of finger tip to have a larger surface area for a less bulky sensor implementation. The flexible sensor measures either backscattered or transmitted light, with the typical measurement site being the top of the wrist ulnar bone.

All devices introduced are hybrids between medical and commercial devices. Some of these hybrids need a system to be set up by a hospital and consist of measuring devices, data storage units (or data centers managed by the company), and tablets (or any other display unit). These points, along with maintenance, training, and handling fees, make these systems’ prices higher than others and not affordable for the general public. Apart from the price range, these comparable to the benchtop medical devices are not, in some cases, a muti-modal sensing device. Having the ability to measure temperature, oxygen level, movement tracking, heart rate, fall detection, and even impedance sensing on a single device is far from commercial implementation and still rare in research. For instance, a multi-modal sensor implemented by Shimmer [28], integrates ECG, respiration, inertial and GPS sensors on a single device. Researchers at the University of South Florida are using this sensor to monitor those who have already tested positive for Covid-19 [29]. In this research, skin temperature, and oxygen saturation are the main symptoms being monitored by a wearable device mounted on the chest. Using artificial intelligence and machine learning on the data collected, patterns that could lead to a better understanding of patient outcomes are extracted. A good example of widely available commercial sensor is the glucometer, a minimally invasive device, now available with the price of less than $10 USD all over the world [30]. A summary of the commercial devices introduced in this section and section III is shown in Table 3.

**TABLE 3:** Commercial Wearables for Body Temperature and Blood Oxygen

Name	Category	Other Measurement	Connection to Body	FDA Cleared	Reusable	Price^*^
TempTraq	Body Temperature	–	Patch	Yes	No	$20
Fever Scout	Body Temperature	–	Patch	Yes	Yes	$45
Oura Ring	Body Temperature	Heart Rate, Steps	Ring	Yes	Yes	$299
FeverWatch^**^	Blood Oxygen, Body Temp	Blood Pressure, Heart Rate	Wrist Worn	No	Yes	$50
Garmin Vivosmart^**^	Blood Oxygen	Activity Tracker,ECG	Wrist Worn	No	Yes	$100
FitBit (Charge, Ionic, Versa)	Blood Oxygen	Activity Tracker,ECG	Wrist worn	Yes	Yes	$120
iHealth	Blood Oxygen	–	Finger Clip	Yes	No	$49
Masimo MightySat	Blood Oxygen	–	Finger Clip	Yes	No	$299
VitalPatch	Body Temperature	Activity Tracker, ECG, Heart Rate, Respiratory Rate, Body Posture, Fall Detection	Patch	Yes	No	^***^
Oxitone	Blood Oxygen	Stress Levels, Sleep Cycles	Wrist Worn	Yes	Yes	^***^

^*^ An average of price based on prices available on internet at time of writing was considered.

^**^ Although only US FDA approved devices are included, Fever Watch and Garmin watch are included due to similarity to FDA cleared rivals.

^***^ These devices are available for medical professionals to use, and the price is estimated to be more than $400 considering the needed platform.

## IV. Sensors

Continuous monitoring of patients in recent years has involved ECG sensor leads connected with wires onto the patient’s body, a nasal cannula, an $SpO_{2}$ clip, and other wired sensors attached to a display close to the bed. These wired sensors, while displaying real-time data, can only be accessed while in the patient’s room. Furthermore, The patient is dealing with the stresses of illness, they are immobile, confined to the hospital bed, and experience a sense of incapacitation. This can cause discomfort and other negative physiological consequences such as deconditioning of the cardiovascular system caused by decreased stroke volume and cardiac output [31].

The integration of wireless with sensors and transducers into wearable systems is becoming more common due to progress in micro and nano-fabrication technologies. Here, sensors with applications in detecting symptoms of COVID-19 are reviewed.

### A. Pulse Oximeters

One of the most significant biological processes in the human body is transporting oxygen by hemoglobin through the circulatory system. The percentage of hemoglobin saturated with oxygen ($SpO_{2}$) is a critical parameter to be measured in patients since lack of oxygen can lead to brain damage, heart failure, and death if it falls below 95% oxygen saturation [32]. Pulse oximetry (used to obtain a photoplethysmogram or PPG) quantifies blood oxygen saturation levels based on the light absorption characteristics of oxygenated and deoxygenated hemoglobin. The principles of pulse oximetry have been known since 1935, when Karl Matthes (German physician 1905–1962) developed the first 2-wavelength earlobe oxygen saturation meter using red and green filters. However, widespread clinical use was not until the early 1980s [33].

**FIGURE 2.**
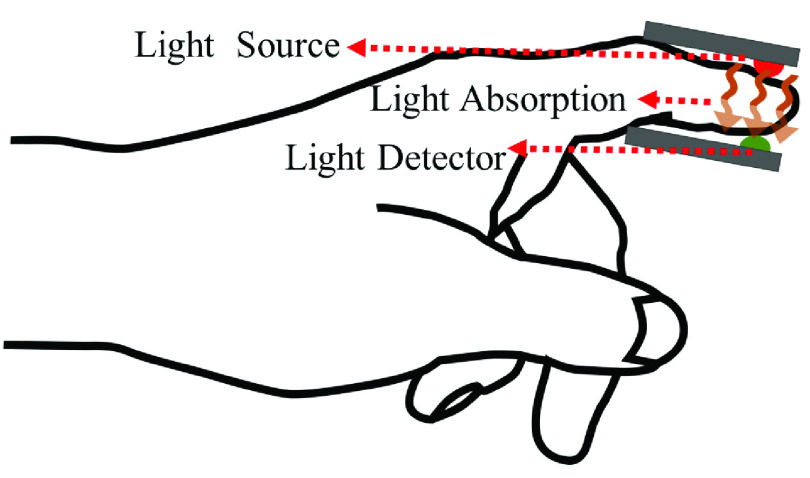
A pulse oximeter emits infrared signal and measures the unabsorbed light.

In pulse oximetry, red and infrared light from light emitting diodes (LEDs), pass through the probing site and the detected light is measured (Fig. 2). Typical measuring sites are the finger, the toes, and the lobe of the ear. Most sensors, however, are located at the finger tip. In pulse oximetry, heart contractions increase the blood flow at the measuring site, leading to increased light absorption. The received waveform at the photodetector contains peaks representing the heart rate.

The infrared light is absorbed by oxygenated hemoglobin. Oxygenated hemoglobin allows more red light to be transmitted through it while Deoxygenated haemoglobin is relatively transparent to infrared light and absorbs red light. The difference in absorption of the two hemoglobin states results in good sensitivity. The detected signal provides information on what proportion of the hemoglobin in the blood is deoxygenated dark red hemoglobin versus bright red oxygenated hemoglobin. The PPG signal consists of an AC component, which is the desired signal and DC component that represents the constant absorption of non-pulsatile tissue within the light path [34], [35]. The AC signal consists of variable light absorption due to pulsating volume of arterial blood.

The DC components are sources of noise and are filtered using high-pass and low-pass filters. The DC component consists of constant light absorption due to non-pulsatile arterial blood, blood in veins and tissue, and bone. The small AC component of the PPG signal is amplified for readout [36]. The advantages of pulse oximeters (PO) are continuous monitoring, accuracy, non-invasiveness, potential cost savings and less stress on the patient [37].

#### 1) Commercial Pulse Oximeters

In the last couple of years, applications that use mobile phones to measure blood oxygen have been published, Ox, Pox, and iOx for iPhone are among the most prominent. These applications use the device’s built in camera to detect $SpO_{2}$. These mobile applications does not use any extra sensors and are convenient in this sense. Limited research has been carried out to validate $SpO_{2}$ obtained from phone applications using healthy volunteers [38], [39] or patients admitted with low blood oxygen in the emergency room [40], with controlled data collected from electronic medical-grade $SpO_{2}$ measurement systems. Most phone applications showed moderate agreement with hospital/medical grade pulse oximeters. However, these applications are not accurate enough to recommend to patients or health providers, even as a personal device. The results in [39] showed that in 20% percent of the cases the results were falsely abnormal for healthy children. Reduced blood oxygen can be a critical symptom and inaccurate results, especially reporting the levels as too high, can cause medical professionals to misdiagnose and under or over treat the patient.

MightySat is the $299 Masims commercial pulse oximeter [25]. Apart from the clinical use, the device is also advertised for in-home use. In addition to the respiration rate, it measures oxygen saturation, pulse rate, perfusion index, and Masimo’s Pleth Variability Index (PVi) [25]. Masimo’s measure-through motion capabilities provide accurate and continuous data in challenging patient conditions. The MightySat weighs about 100 grams and is powered through 2 AAA batteries (included in the weight). The device is capable of doing 1,800 measurements before it needs the battery changed. It also facilitates monitoring and sharing of measurements via Bluetooth.

Another FDA approved pulse oximetery device, much cheaper than the former device is presented by Ihealth [41]. The PO3M is a non-invasive wearable device that can detect the oxygen saturation of arterial hemoglobin ($SpO_{2}$) and pulse rate. This wearable fingertip device is indicated for adult patients in home and hospital environments (based on the FDA document). It detects the effects of activity on oxygen levels and pulse pre- and post-workout (based on their website). The device uses 3.7 V Li-ion, 300 mAh battery, and weighs 180 grams. It has Bluetooth connectivity to transmit measured data to the device.

#### 2) Pulse Oximeters in Research

A photoplethysmographic sensor is capable of monitoring critical vital signs such as heart rate (HR) and blood oxygen saturation ($SpO_{2}$). With pulse oximeters usually being a module in a system that measures multiple physiological signals, power consumption is the primary constraint. In 2013, the first fully-integrated pulse oximeter was designed by Konstantinos N. Glaros [42]. Adequate signal to noise ratio (SNR) to detect changes in $SpO_{2}$ levels and low power consumption are some of the advantages of this new design. In his design, Glaros used a switched-integrator as a noise-limiter, and a high-pass filter plus a switched large bias current to lower the transimpedance amplifier’s thermal noise [42].

Even in on-chip pulse oximeters, two LEDs are flashed alternatively, dominating the system’s power requirement. To reduce power consumption, chopping, reduced duty cycles, and high switching frequency are frequently used. However, the signal to noise ratio, and thus the accuracy of the sensor, is reduced by smaller duty cycles. Trying to control the duty cycle of the LED, turning off modules of the sensor, and sampling the signal where the useful information lies (near the peaks) have been among the methods to reduce the power consumption [43], [44]. In [45], all system components work synchronously on the applied duty-cycling using a non-uniform pulse stream. This method reduces the diode driver’s power up to $30\times $ while retaining most of the information. However, this design realized the needed filters with large resistors, contributing to additional circuits and increased power consumption.

In [46], the low-pass filter is removed and DC level detection and calibration is implemented using a current mode digital to analog converter (DAC). However, the slow frequency of updating the measured DC resulted in signal errors from motion artifacts and ambient light interference. Reference [47] achieves one of the best power dissipation figures using aggressive duty cycling and input signal-aware adaptive sampling. However, the advantage of minimum input-referred noise is offset by increased high pass frequency and severely reduced gain. In [48], power and area is reduced by weakening the requirement for the sensor’s dynamic range. It is accomplished without a DAC, simplifying the design. To estimate the performance quality of the design, heart rate and $SpO_{2}$ algorithms are calibrated. To maintain a good SNR and reduce the LED power significantly, [49] uses a high sensitivity photodetector with ultra-low noise and low power readout system. Quasi floating gate technology, inverter cascode techniques, and pseudo resistors implement the filters used in [50] to achieve low-power and high sensitivity. This design has not yet been fabricated, however floating gate designs are one of the promising areas that improve pulse oximeters. In [51], automatic gain control is used to reduce the power consumption. When the photocurrent is very large, the gain control halves the gain of the amplifying blocks. This mitigates distortion of the PPG signal. The design also uses a background light cancellation loop to reject the input DC photocurrent, reducing the noise.

Table 4 compares the power consumption of the mentioned pulse oximeters and indicate the systems targeting lower power consumption. The power consumption of the system, without the current needed for driving an LED, is still in the range of few ten micro-watts, making this module one of the most power-consuming modules in a system of sensors. New research goes as low as $4.6~\mu \text{W}$, including the ADC for an accurate pulse oximeter [49].

**TABLE 4:** Blood Oxygen Saturation Devices Implemented Using CMOS in Research

	[42]	[44]	[45]	[46]	[47]	[48]	[49]	[51]
Technology ($\mu m$)	0.35	0.18	0.18	0.18	0.13	0.18	0.18	0.35
Supply Voltage (V)	3.3	1.8	1.2	3.3/1.8	1.5	2.5	3.3/1.8	2–3.7
Oximeter Type	Transmission	Transmission	Reflective	Reflective	Reflective	Reflective	Reflective	Reflective
Sampling Frequency (Hz)	100	165	4	0.5,1,2,4	100	100	40	Continuous
LED Bias Current (mA)	7.1	25.6	0.036	0.0033	50	50	9.43	3 mW
Size (mm^2^)	1.265	1.84	10	8.45	10^c^	2.25^c^	20	0.64
Power Dissipation ($\mu \text{W}$) ^a^	363	216^b^	171^b^	$99^{a}$	68.7^b^	180	4.6^b^	14.85
LED Duty Cycle (%)	4	0.7	0.0125	N/A	20	25	0.07	Continuous

^a^ LED Power is excluded.

^b^ ADC power is included.

^c^ Estimated value.

### B. Temperature Sensors

**FIGURE 3.**
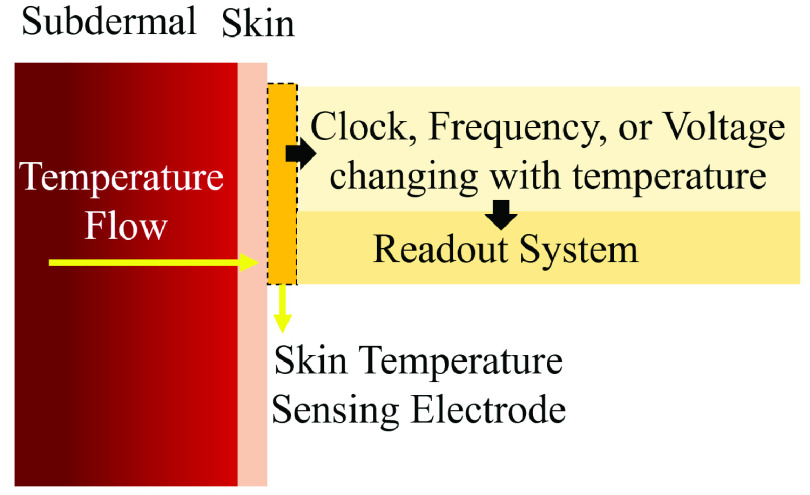
Skin patch temperature sensing system.

Measuring body temperature in clinical practice is immensely valuable for diagnostics. While in most cases, just the presence or absence of fever is really significant, continuously measuring temperature can offer us unprecedented insights into the cause and nature of a fever and help provide better and more responsive care. An overview of a wearable temperature sensor is shown in Fig. 3. Generally, the fever measuring system has a voltage, clock, or frequency that changes in a predictable manner with the temperature. This change should be compared with a reference signal to extract the variation. This variation caused by the difference of the fever temperature from the normal temperature is then translated to digital data, frequency or other means to be transmitted.

Recording trends in body temperature over time allows us to understand the intensity and progression of a fever reliably. This data can also enable a physician to precisely follow the fever’s onset and prognosis. Fever has been the common element that has been repeated in every pandemic. In COVID-19 fever is very common making temperature sensors a critical component of a wearable sensing system.

#### 1) Commercial Temperature Sensing Systems

Since temperature sensing is very common in infants, there are plenty of commercial temperature sensing systems available, however, here we have only considered the devices that can be used for adults as well as infants and had a means of reading the data in a portable manner (e.g. sending the data to mobile phone application). TempTraq [52] is a single-use device that is a soft, comfortable patch that continuously monitors body temperature for up to 48 hours. The patch sends the reading as well as alerts to compatible mobile devices via Bluetooth. TempTraq is purposed to monitor the patient’s temperature without disturbing them.

Fever scout by VivaLNK [53] is a multi-use wearable thermometer patch that is worn under the arm, where it continuously monitors body temperature. The measured temperature can be sent up to 100 feet away to a smartphone through the accompanying app and the patch is 41 mm by 61 mm. This patch is purposed for post-operation fever, flu infections, child fevers, and drug effects. The device is water resistant and can last for 1 week on a battery charge. The chance of cross-infection increases as caregivers typically have to record a patient’s temperature at prescribed intervals throughout the day. Since many people can be asymptomatic, it is possible for caregivers to be carriers further transmitting the disease. VivaLNK updated their system for the current pandemic and has developed a continuous monitoring and alert solution to automate continuous fever tracking. Using trends and thresholding, caregivers have access to patient’s temperatures through IoT enabled medical wearable temperature sensors connected to a central monitoring system. The medical staff can then identify the patient and can respond accordingly.

Another interesting commercial device that includes a temperature sensor is the Oura ring. The device is worn around the finger and performs body measurements such as heart rate, temperature, and step counts [54]. This ring can last up to 7 days per charge, is water resistant and weighs only 7 g. The ring shows the detected data on a mobile application, communicating by wireless signals.

#### 2) Temperature Sensing Systems in Research

There are various low power temperature sensors presented in recent literature. Many well-known designs use bipolar junction transistors (BJTs) where the emitter to base voltage has a linear decline with an increase in temperature. BJTs are typically not used as temperature sensors for miniaturized systems due to the power consumption being, typically, in the $\mu \text{W}$ range. Proportional to absolute temperature (PTAT) voltages can be generated from two BJTs. However, these sensors need a high accuracy ADC to achieve accurate temperature sensing [55]. In [56], a $\Sigma \Delta $ modulator is used as an ADC because of its high resolution at lower frequencies. Different topologies of $\Sigma \Delta $ modulators have been considered in temperature sensors. The requirement for operational transconductance amplifiers (OTAs) in $\Sigma \Delta $ modulators make them bulky and power-hungry. Many designs try to relax the bias issue of $\Sigma \Delta $ modulators by utilizing self-biased OTAs. However, even with this, the power consumption can be a few orders of magnitude higher than the typical sub-$\mu \text{W}$ target.

In order to compensate for CMOS’s process variation, accurate ADC’s will typically have large power consumption and large area. The majority of sensors designed using this method showed better than $1{^\circ }\text{C}$ accuracy and a 5-$100~\mu W$ of power [57]–[59]. Reference clocks along with frequency circuits that are dependent on temperature have also been used as temperature sensors. These have the advantage of less power consumption but give up resolution and accuracy [60]–[63].

Most of these designs use an external clock, which may not be convenient for portable devices. Other designs [64]–[66] demonstrate clock-less temperature sensors and convert the measured temperature to a delay or pulse width with the advantage of low power consumption. In [67] a current that is dependent on temperature is generated. This output current, ideally proportional to temperature, then uses a current controlled oscillator to translate the temperature dependent current value into a frequency. The oscillation frequency is proportional to the PTAT current. Any nonlinear error can be mitigated through calibration (eg. two point calibration). However, having a good calibration system can increase the accuracy at the cost of area and power consumption.

Weak inversion MOS devices, instead of BJTs, can enable ultra-low-power operation in the 10’s to 100’s of nW. Designs such as [68], set the goal of having a temperature sensor with power consumption of sub $\mu \text{W}$, with power consumption of 220 nW at room temperature with continuous operation. The work in [69] uses dynamic threshold voltage MOSFET transistors and achieves 600 nW for the power consumption. However, having any MOSFET that uses specialized steps in the fabrication process increase chip costs. By modifying the voltage-to-current conversion module and the current mirror structures, 71 nW was achieved by [62]. Using serially connected sub-threshold MOS transistors a power consumption of 119 nW was achieved by [64]. Biasing two MOSFETs in weak inversion at different $\text{V}_{DS}$’s and using the ratio of the currents [70] a linear approximation of the exponential function was realized with only 11 nW power consumption.

In applications that have a limited temperature range and small variations (e.g. dynamic memory, implantable devices), accurate measurements for every temperature are not necessary. In [71], a folded temperature sensor was proposed, where accurate measurement was done by moving to a different range. This segmented temperature sensing leads to a reduction in supply voltage. The sensitivity and limit of each section can be changed, by an alignment of these segments. Table 5 shows the many temperature sensors, designed in research that can be used in wearable systems. Conversion to frequency or the digital domain can offer advantages such as improved compactness and power consumption [60], [64], [66], [72]–[74]. Having a BJT-less temperature to frequency/digital structure can take advantage of having a low power design, by use of sub-threshold MOSFET transistors and removing the need for external clocks and power-consuming ADCs.

**TABLE 5:** Temperature Sensor Comparison

Reference	Operating Voltage $(V) $	Accuracy ($^\circ C $)	Power $(nW) $	Resolution ($^\circ C $)	Area $(mm^{2}) $	CMOS Technology ($\mu m $)
[60]	2.1	±1	110	0.035	0.084	0.35
[69]	0.85	±0.4	600	0.063	0.085	0.16
[62]	1	±1.5	71	0.3	0.09	0.18
[63]	–	−0.84/+0.34	305	0.294	0.002	0.18
[64]	1	±0.8	119	0.21	0.0416	0.18
[65]	1	±1.5	350	0.3	0.14	0.18
[66]	1	±0.8	405	0.3	0.0324	0.18
[73] ^*^	0.6	0.47	117	–	0.96	0.18
[67]	1.1	2	150	–	0.0014	0.13
[56] ^*^	1	±0.25	27800	0.18	0.0033	0.065
[70]	0.8	−0.9/1.2	11	–	0.074	0.18
[75] ^*^	0.8	−4.2/+3.82	144	2.14	0.0016	0.029
[74]	0.5	±0.2	290	0.2	0.02	0.18
[76]	0.5	± 0.15	195	0.2	0.008	0.13

^*^ Sensors marked with asterisks are not fabricated, simulation results are included.

### C. Impedance Sensors

**FIGURE 4.**
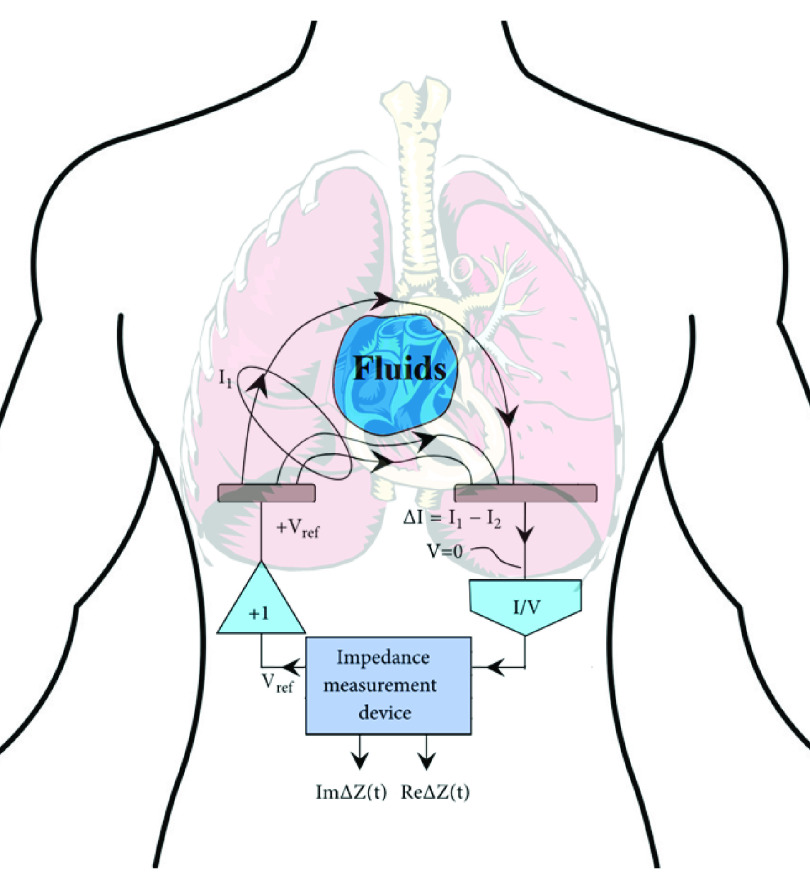
Trans-thoracic impedance sensing method for measurement of fluid buildup in lungs.

Many different biomedical applications have been implemented using electrical impedance measurement of biological tissues. This measurement relies on the modulation of the electric field between two conductive electrodes. The physical shape and location, presence and absence, and density of a tissue (or cells, substances, chemical particles, and elements) between these two electrodes will result in a variation of the applied electric field. This modulation of the field based on the tissue between the electrodes changes the measured current compared to when there is no tissue between the electrodes. This method is shown in Fig. 4. Using a small AC source that is not noticeable by humans, this process is classified as a non-invasive procedure and is considered a promising clinical monitoring technology. Impedance measurement is also cheap and straightforward to implement, making it an interesting solution for remote monitoring of physiological parameters in wearable systems. Bio-impedance measurements have applications in body composition measurement in sports, firefighting, military, medicine, respiration analysis, and blood flow [77], [78]. As an example, hemorrhagic shock and hypotension caused by blood circulation in the brain is a significant cause of death [79]. Management of obesity and monitoring fluid changes while physically active is among other applications of bio-impedance sensing [80], [81]. However, for pandemic resolution, the devices would be used for cardiac monitoring and pneumonia detection [82]–[85].

Fluid build-up is an early sign of heart failure. The range of fluid levels and fluid pressures are small. The fluid moves from capillaries into the interstitial space, then returns to the circulation by the lymphatic vessels. The balance between hydrostatic and the osmotic pressure in the blood results in the overflow from the capillaries. Particle concentration in interstitial end of the plasma reduces while there is an increase in the overflow from the capillaries. To balance the outflow, fluids build up in the tissue while sensing any differences outflow between the hydrostatic pressure and osmotic pressure. The fluid causes swelling in the tissue and reduces the tissue functionality. If the build-up occurs in the lungs, then it results in breathlessness [86]. Other parameters, such as an increase in pulmonary circulation, will affect the outflow of fluid from the capillaries. With an increase in interstitial fluids, the lungs become more inflexible and heavier, which also leads to breathlessness. Systematic circulation controls the amounts of fluids that can be buffered in the tissues [86]. In COVID-19 the lungs are ground zero of disease, therefore monitoring the lungs and the fluid accumulation in the lungs can be a critical part of a respiratory pandemic detection and resolution.

#### 1) Commercial Thoracic Impedance Measurement

To the author’s knowledge, there have been no commercial FDA approved impedance sensors for trans-thoracic impedance measurements. In 2009 the FDA cleared a wireless, non-invasive cardiac monitor system developed by Corventis Inc. [87]. The sensor collected patient data and transmitted it to a secure website where clinicians could potentially act on the data. The device was, however, later modified to be used for implantable cardiac monitoring.

#### 2) Thoracic Impedance Measurement in Research

A review of sensors with applications only in thoracic impedance measurement is presented in this section. Having an implantable impedance measurement device is the primary clinical method to measure thoracic impedance. However, having invasive implantable devices is not applicable when there is a surge of influenza patients. There is thus much ongoing effort into developing trans-thoracic electrical impedance measurement systems that are wearable and portable. In these wearable devices, patches and textile can be used for the electrodes. In [83], a low-power, low cost, wearable, high resolution cardiac healthcare system used a patch sensor to monitor thoracic impedance and ECG. This adhesive poultice-like plaster has 25 silver ink based electrodes and was fabricated as a 15 cm $\times15$ cm 4-layer patch. The impedance in series with the current injection leads is increased due to the dry interface between the skin and the electrode in patch and textile electrodes. In [84] the electrodes for thoracic impedance are an integral part of a T-shirt. Textile material, that is also stretchy, with better contact have also been used [85].

There are also micro-controller based portable or even wearable systems to detect thoracic impedance [88]–[92]. These microcontroller based devices are being replaced with integrated systems to reduce the size, weight, and power of the system.

There are many impedance measurement topologies available. Conversion to frequency [93], [94] and extracting the real and imaginary part of the impedance with a lock-in amplifier [95]–[97] are among the typical topologies. The measurement principle of the first method is to obtain a frequency proportional to impedance using a periodic bi-directional current across the impedance. The signal characteristics of potential are charged on the electrode, which is then translated into frequency, enabling the readout system to extract the tissue impedance between the two electrodes. In the second method, the complex impedance is calculated by using the in-phase and quadrature signals of the interrogating signal. This method sweeps the frequencies of the sinusoidal signal applied to the electrodes. The magnitude and phase are obtained using low pass filters. An illustration of portable systems for thoracic impedance measurement in state of the art research is shown in Table 6. As is evident by the table, there have been a few impedance measurement systems available that measure an impedance range of few $\Omega $ to a few k $\Omega $ with errors smaller than an $\Omega $. These devices already match the clinical-grade demands in terms of accuracy for impedance measurement. With a small area in most of these devices, the main problem remains to be power consumption. The significant portion of the power consumed in the systems is not the sensor itself, but the data transfer means utilized to send the impedance such as the RF Modules and Bluetooth transmission. Using an on-chip means of data transfer can therefore reduce the power consumption by orders of magnitude.

**TABLE 6:** Comparison of Thoracic Impedance Measurement Systems

	Range ($\Omega $)	Sensitivity ($\Omega $)	Hardware Implementation	Power (mW)	Size (mm$\times $mm)	Weight (g)	Data Transfer
[83]	10–10 k	0.1	180 nm	3.9	$150\times 150$	N/A	SRAM
[85]	10–20	3	Discrete	546	$145\times 40$	30	Bluetooth
[89]	1–1k	0.1	Discrete	406	$85\times 65$	200	Bluetooth
[90]	10–40	0.01	Discrete	120	$38\times 18$	70	Serial
[91]	1–54	0.5	Discrete	14	$48\times 30$	N/A	RF module
2.4GHz nRF24L01
[92]	1–54	0.5	Discrete	N/A	N/Z	N/A	RF module 2.4GHz nRF24L01
[95]	1–1k	0.001	350 nm	100	N/A	N/A	N/A
[98]	1–10k	1	130 nm	3	$45\times 25$	12.3	RF module 433MHz QAM-TX2

## V. Discussion

Pandemics have been occurring throughout the history of humankind. In the last 100 years, we advanced from the literacy rate of 23% to 86% [99]. At the same time, technology moved from the toggle light switch to artificial intelligence. Advancements in medicine have pushed life expectancy from 46 years old in 1900 to 72 years today [99]. The process of testing for diseases has come a long way from symptom-based diagnosis to portable lab tests with the ability to detect viruses in a few minutes. For COVID-19, Cue Health Inc., a healthcare technology company was awarded a $13 million (USD) contract to develop a test for the COVID-19 causing virus [100]. Vaccine production has changed tremendously in the era of genetic engineering. However, because of the increased standards and various regulations on their production, the process of having a vaccine mass-produced still takes about 9–18 months. Generally, the process of controlling a pandemic has not changed significantly from past pandemics, going back to the Spanish flu. The much-expected vaccine will most likely be available after the disease’s peak has passed, and apart from self-quarantining, there are not many more guidelines. The traditional model-based approaches and simulations, though extensively developed, cannot adequately contain the real time nature of epidemics. These methods may lack timely data and may provide inaccurate predictions since it depends highly on assumptions on the behaviour of a new virus [101]. There is no doubt that modern hospital equipment are science-fiction-like to people living in 100 years ago. However, these state-of-the-art medical equipment are still limited when there is a patient surge which can block the access for other critical patients reaching timely proper care [102].

To ease the surge of patients, other means of technologies should be utilized to monitor patients. Impressive sensors are being developed in research labs at companies and in academia. There have been various efforts to incorporate technology in the struggles of the modern world with COVID-19. Giants of technology (Amazon, Facebook, and Google) met to propose tools and collaboration preventing misinformation [103]. Microsoft has stepped into developing a chat-box to screen patients before they donate plasma in-person to help with clinical research and treatment. Apple and Google have committed to develop contact tracing functionality based on bluetooth as part of the underlying operating system. A more detailed tracking system was implemented by China to keep the spread of COVID-19 in check. The app, however, requires personal location tracking and questions and was described as invasive and hard to implement in any other country. In [104], data gathered from 200,000 Fitbit users was extracted to predict influenza-like-disease epidemics at the state-level based on sleep cycles and resting heartbeat. In this experiment researchers concluded more sensors (temperature and respiratory measurement) would be an improvement. changemarkerIn another study, heart rate data from active tracker wearers (e.g., Fitbit, Apple Watch, Garmin, Amazefit, OURA, Beddit) are detected to provide early indication of influenza-like illnesses [105].

There are many other interesting sensors for preventing or monitoring pandemic events. A smart ring is presented in [106] that implements a discrete inexpensive method for real-time monitoring of hand hygiene. In this ring, an electrochemical fluid sensor detects when the hand makes with water and indicates the required time for hand-washing using an LED. The sensor is capable of identifying different types of hand sanitizers (foam, liquid, alcohol based) and appoint different hand washing times for the different sanitizers. In another research at Northeastern University [107], a flexible, soft sensor is worn at the base of throat (on the visible dip) to quantify the intensity of the cough and potential patterns. Despite these interesting achievements, sensors that monitor vital signs are still the main tool to monitor a pandemic.

Though a wearable monitoring system for vital signs is necessary to have in the event of a pandemic, a general influenza patient monitoring system can save thousands of lives every year. According to WHO [108], seasonal influenza kills up to 650,000 people every year, with 70 to 85 % in persons with pre-existing conditions that are older than 65 years. In the 2017–2018 season alone, 80,000 people died of seasonal flu complications in the USA, with long term nursing homes and senior care facilities being a hit both in the seasonal flu and current COVID-19 pandemic [109]. In 2007, [110] indicated that an influenza pandemic could be fatal and out of control in long-term care facilities, and an epidemic can have severe consequences in these communities. In 2020, indeed care facilities are among the challenges of pandemic control. Apart from older adults, young children, pregnant women, and those with chronic issues are at higher risks of complications due to the flu. Having a wearable system to monitor patients can save lives every year, reduce unnecessary hospital admissions that can be inconvenient and expensive for people who live in countries without a public health system.

Over the last couple of years there has been some advancements in studies involving wearable devices from companies. Companies mostly consist of startups and small businesses scattered around the world, with research areas that is far from each other. The difference in the focus of wearable devices, grants the advantage of providing different views on a single problem. Different numbers of sensors integrated into a single device from companies like Masimo, Vital Connect, and Oxitone, show the feasibility of having a single device with many sensing modalities and the necessity of having a cloud data center for professionals to have easy, online access to the data. These portable sensing systems are not widely available in the current influenza pandemic. In the world of commercial FDA cleared health monitoring wearable devices, which is a rapidly growing, there is no wearable device capable of sensing common symptoms of influenza that has affected respiratory system. The lack of such a system is because of the many challenges in the design of a wearable multi-sensor system. These challenges include powering the devices, design of wearable electrodes, secure transmission of data, and fabrication.

### A. Powering the Wearables

As wearables go smaller in size and become more widely adopted, one challenge for these devices is energizing them. Circuits designed in newer technologies are in the $nW$ realm of power consumption, letting the whole system consisting of several sensors to stay in $\mu W$ realm. A $300~\mu \text{W}$, 1.5 V sensor can work for up to 6 months with a simple 1000 mAh coin cell battery that is 24.5 mm in width and 7.7 mm in height and weighs 10 gram. With on-chip Bluetooth systems going toward $\mu W$ [111], [112], a system can be implemented on-chip using small CMOS technology and be powered up with batteries, lasting days. However, conventional methods of using batteries cannot provide the robust reliability and flexibility needed in some wearable devices.

Batteries are too bulky for devices such as smart contact lenses or patches, and the idea of charging them is not viable for devices that need to worn for a long period of time (e.g. heart rate monitoring systems). Thus, energy harvesting is a potential means to eliminate the need for batteries in wearables. Energy harvesting from ambient light, radio frequency, thermoelectric, and human powered have all been tried and their potential showcased [113]–[116]. From the 1980s, solar power has been harvested and stored in photovoltaic (PV) cells to power up calculators and wrist watches. These can be implemented with a flexible design on the body [117], [118] or under the skin [115], [119], [120] to provide $100~\mu \text{W}$ to 100 mW per $cm^{2}$ based on the light intensity. Though these devices have rather high power intensities, they are not continuous since they depend on the environment.

Thermoelectric is another method that has been used since the 1990s [121] and can provide $60~\mu \text{W}$ per $cm^{2}$ if there is a temperature delta of $5~^\circ \text{C}$ to $10~^\circ \text{C}$ from the surrounding environment to the human body [122]–[124]. Another widely available source of energy is RF waves, a seemingly ubiquitous source due to the prevalence of difference frequencies of wireless technologies. This method is widely available making power harvesting easier. However the power density is slightly less, $1~\mu \text{W}$ per $cm^{2}$ for ambient source [125]–[127] and $15~\mu \text{W}$ per $cm^{2}$ for external source [113], [128], [129], showing the dependence of power to the distance from the source.

In recent years harvesting energy from human bio-fluids have attracted a lot of interest. The energy from food consumption is converted into chemicals and kinetic energy, and the idea is to use this energy to power up wearables [130]. Piezoelectric power harvesting from kinetic energy and glucose/sweat fuels cells have the potential to provide power densities in the range of $200~\mu \text{W}$ to 1 mW per $cm^{2}$ [116], [131]–[133]. Though this method is most promising, power density levels depend on the level of activity and differ from person to person and still need improved fuel cells. In terms of circuit design all mentioned approaches require a converter or transducer device that converts energy to a power usable for the sensor and a power management module to regulate and monitor the power for the sensor. For energy sources that are not constant, such as ambient light, heat, human movement, and bio-fluids, another module needs to be added to store the energy. This will contribute to the area and complexity of the circuit.

### B. Electrode Design

Another challenge is the electrodes design. Conventional electrodes are silver/silver chloride (Ag/AgCl) adhesive button electrodes that need a conductive gel to ensure good conductivity. This gel inevitably leaves residues, making their use uncomfortable. This conduction gel dries quickly and is not suitable for long-term monitoring. With a dry interface, the motion artifact affects the results immensely due to friction and slipping of the electrodes [134].

Stretchy textile materials for better quality contact have also been implemented [84]. These may however prevent a challenge in usability and comfort for older patients. The design of [85] uses electrodes embedded in a shirt to measure thoracic impedance. This electrode also is grouped into the dry interfaces with considerable inaccuracy when motion artifacts are taken into account. There is the possibility of using slightly invasive—but painless—wearables in the near future. These approaches include flexible stickers full of micro-needle sensor arrays that can track chemical changes just under the skin. An example application detects the presence of an enzyme using a bio-marker on the skin surface, particularly within skin moles for the rapid screening of skin melanoma [135]. Newer cheaper, robust electrodes that can be worn for a long period of time (preferably days to weeks) and work accurately despite the patient moving need to be developed commercially to get a step closer to a wholesome wearable patient monitoring system.

### C. Security and Encryption

Another design criteria is the secure transmission and storage of information. Home health monitoring is not new [136]. However, the infrastructure needed to support these connected devices is an active area of research with significant challenges remaining. Groups such as the Personal Connected Health Alliance and HIMSS indicate policies that ensure ease of access, interoperability, and security of data. In contrast, industry views security from a business perspective. This has resulted in slow adoption of standards and mechanisms for data sharing. However, it is clear that wearables are one of the best tools for mitigating the effects of future pandemics. A successful example is, [137], where for controlling the mosquito borne Chikungunya virus, a healthcare system based on a wearable IoT sensor was proposed. The system focused on the use of intelligent fog and cloud computing to improve information acquisition and storage. Fog computing is decentralized cloud computing, where storage devices are implemented between the data source and the cloud. However. this research is done without experimental data from a wearable system, instead, basic vital symptoms data is generated. Another example is the design and deployment of large-scale applications for body area networks based on cloud computing platforms. Reference [138]. This architecture, however needs to be integrated with data analysis tools to have a complete system. Therefore, having a data center to store the data is another complication of a portable wearable system that can be probed from various sides.

### D. Cost

A complete low-power, portable sensing system for patient monitoring fever, SpO_2_, thoracic impedance mentioned, along with respiration rate exist in research. In terms of price, chip production is a pricey process, with technologies going smaller. Currently 65 nm or smaller technologies can cost up to a few hundred thousand dollars [139]. Non-recurring engineering (NRE) costs, such as teams, equipment resources, and materials other than the ICs, can add up to a couple of million dollars. However, IC’s produced in the range of a million parts will divide the cost to the number of IC’s produced. The costs of fabrication processes and NRE costs to build commercial IC’s is therefore divided by millions. Commercial IC’s price, produced in millions, doesn’t go higher than a few dollars.

Different sensors and actuators can be driven by nano-technology advances [140]. These may be commonly implemented using carbon or gold particles. In the case of implementation with gold particles the price is more expensive than CMOS fabrication with each gram of particles costing as high as $80,000 [141]. Newer methods of implementation of nano-devices with carbon however have a reduced price since the price can be as low as just $15 to $35 for each gram [142]. Apart from the sensor fabrication cost, electrode fabrication must also be taken in to account. electrodes implemented in fabrics, textiles and patches are not expensive [83]–[85], more accurate electrodes implemented with nano-materials can add to the cost of the wearable device. To have a system that can be mass-produced, and gear that can be worn by thousands to millions of people the cost of the entire system should not go higher than a few dollars.

### E. The Ideal System

**FIGURE 5.**
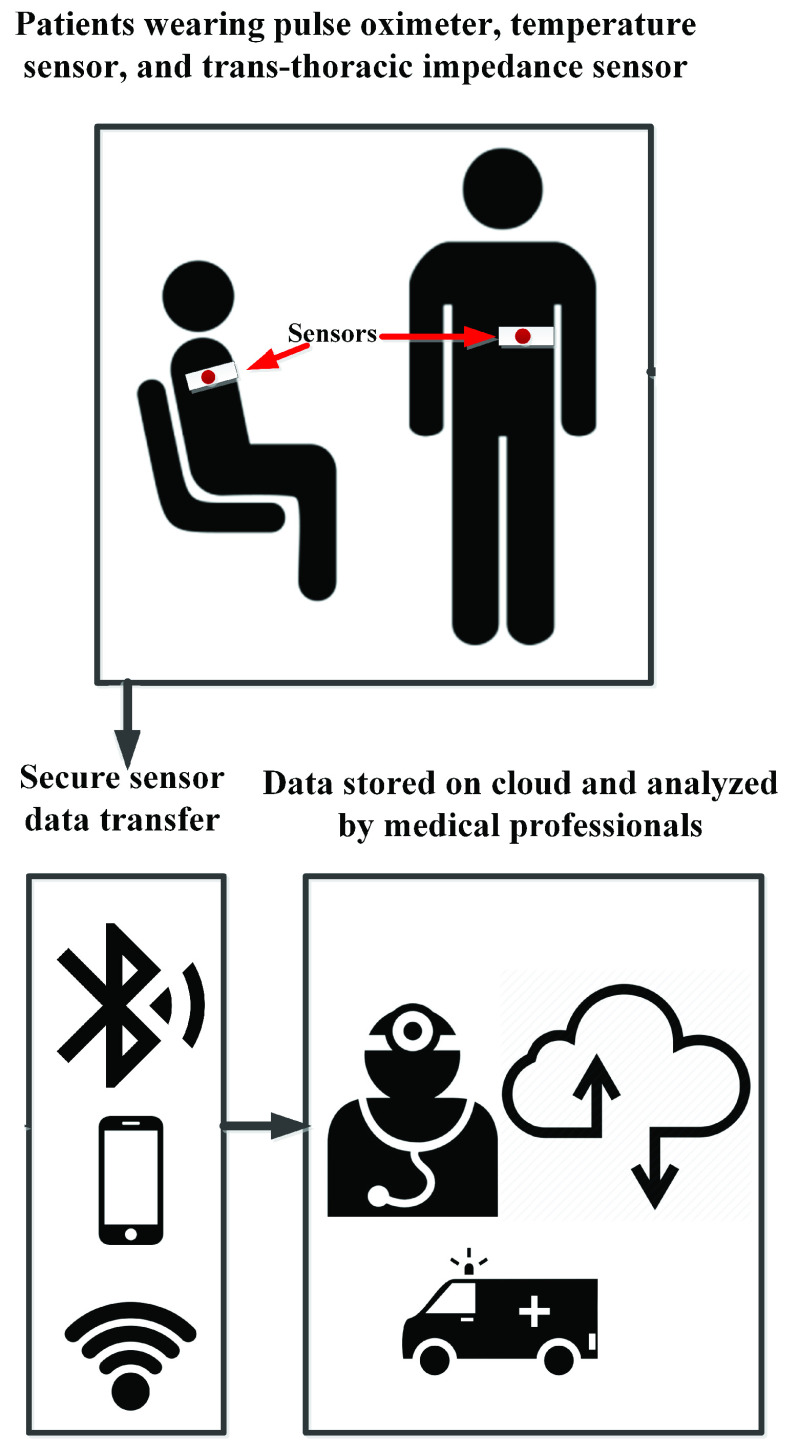
An ideal system for patient monitoring with influenza like symptoms.

An ideal system, shown in Fig. 5, consists of a temperature sensor to detect fever, the basic symptom of influenza and infections, pulse oximeter to measure the blood oxygen saturation, and a trans-thoracic impedance measurement sensor to detect the critical stage of influenza, inflammation in lung, and pneumonia. Other sensors such as ECG, stress level, and sleep cycle detection can also be added to this wearable sensing system to detect more data. Besides sensors, smarter wearable sensors can have a brain of their own processing the data before transmitting them. Having on chip processors, with controlling unit and memory, that does not consume power in range of $10~\mu \text{W}$ to $100~\mu \text{W}$ have attracted a lot of interest recently [143]–[147].

All wearable sensors need a wireless mean of data transfer to send the gathered data from the sensor securely. Generally, off-chip RF or Bluetooth modules are the most power-consuming blocks of any wearable/sensor system. With more than a hundred times more power consumption than $\mu \text{W}$ sensors, having a low-power wireless sensor fabricated on-chip will eliminate the need for power-consuming data transfer blocks. The data is transferred to a data center or cloud to store the data and for the data to be analyzed by the medical personnel and decide the right step for the patient. The right step for the patient can be to stay home and continue the remote monitoring, or to come to the hospital in case the symptoms have gotten worse. This provides a more comfortable environment for the patient to be monitored, mitigating the expensive cost of hospitalization, preventing hospitals from overflowing with patients with mild symptoms, and potentially reducing patient stress.

## VI. Conclusion

The pattern of a Coronavirus species or other viruses causing severe respiratory diseases with influenza like symptoms have occurred for centuries. In pandemics of the last 100 years, the virus itself may have been novel, but the patterns have been repeating. This implies that there are going to be other pandemics in our future. The symptoms of all respiratory disease pandemics are fever, chest congestion, trouble breathing, and muscle or joint pain and complications occur when the blood oxygen saturation level falls leading to lack of oxygen or when fluids accumulate in the lungs. Commercial devices exist that can detect most of the symptoms and research has developed low power wearable sensors and systems in all of these areas. With technologies moving towards lower power and smaller devices, multi-sensor wearable devices that are capable of monitoring patients showing epidemic disease symptoms, storing the data and enabling access for medical personal are now feasible. Having the sensors fabricated in large quantities reduces the price of the sensor. However, there are still a number challenges to overcome before these sensors are approved for clinical use, including improved electrodes and security issues along with design tradeoffs in power, size, and accuracy.
